# Atypisches Vorhofflattern: Diagnostik und Therapie

**DOI:** 10.1007/s00399-022-00887-3

**Published:** 2022-08-19

**Authors:** Marc Kottmaier, Felix Bourier, Sonia Busch, Philipp Sommer, Tilman Maurer, Till Althoff, Dong-In Shin, David Duncker, Victoria Johnson, Heidi Estner, Andreas Rillig, Leon Iden, Roland Tilz, Andreas Metzner, K. R. Julian Chun, Daniel Steven, Henning Jansen, Amir Jadidi, Christian Ewertsen, Tilko Reents

**Affiliations:** 1 Abteilung für Elektrophysiologie, Deutsches Herzzentrum München, Technische Universität München, Lazarettstr. 36, 80636 München, Deutschland; 2 Medizinische Klinik, Klinikum Coburg GmbH, Coburg, Deutschland; 3 Klinik für Elektrophysiologie/Rhythmologie, Herz- und Diabeteszentrum NRW, Ruhr-Universität Bochum, Bad Oeynhausen, Deutschland; 4 Klinik für Kardiologie, Asklepios Klinik St. Georg, Hamburg, Deutschland; 5 Med. Klinikum Kardiologie u. Angiologie, Charite – Universitätsmedizin Medizin Berlin, Berlin, Deutschland; 6 Arrhythmia Section, Cardiovascular Institute (ICCV), CLÍNIC – University Hospital Barcelona, Barcelona, Spanien; 7 Klinik für Kardiologie, Herzzentrum Niederrhein, HELIOS Klinikum Krefeld, Krefeld, Deutschland; 8 University Faculty of Health, Center for Clinical Medicine Witten-Herdecke, Wuppertal, Deutschland; 9 Hannover Herzrhythmus Centrum, Klinik für Kardiologie und Angiologie, Medizinische Hochschule Hannover, Hannover, Deutschland; 10 Klinik für Innere Medizin, Universitätsklinikum Gießen, Gießen, Deutschland; 11 Medizinische Klinik und Poliklinik I, LMU Klinikum der Universität München, München, Deutschland; 12 Universitäres Herzzentrum Hamburg, Universitätsklinikum Eppendorf Hamburg, Hamburg, Deutschland; 13 Klinik für Kardiologie, Herz- und Gefäßzentrum Bad Segeberg, Bad Segeberg, Deutschland; 14 Sektion für Elektrophysiologie, Medizinische Klinik II, Universitäres Herzzentrum Lübeck, Universitätsklinikum Schleswig-Holstein (UKSH), Lübeck, Deutschland; 15 Universitäres Herzzentrum Hamburg, Universitätsklinikum Eppendorf, Hamburg, Deutschland; 16 Cardioangiologisches Centrum Bethanien – CCB, Frankfurt, Deutschland; 17 Abteilung für Elektrophysiologie, Herzzentrum der Uniklinik Köln, Köln, Deutschland; 18 Elektrophysiologie Bremen, Bremen, Deutschland; 19 Klinik für Kardiologie und Angiologie, Abteilung für Elektrophysiologie, Herzzentrum Freiburg Bad Krozingen, Bad Krozingen, Deutschland; 20 Klinik für Innere Medizin – Kardiologie und konservative Intensivmedizin, Vivantes Klinikum Am Urban, Berliner-Herzrhythmus-Zentrum, Berlin, Deutschland

**Keywords:** Herzrhythmusstörung, Atriale Tachykardie, Reentrietachyarkdie, Katheterablation, Antikoagulation, Cardiac arrhythmia, Atrial tachycardia, Reentry tachycardia, Catheter ablation, Anticoagulation

## Abstract

Im Gegensatz zum typischen Vorhofflattern handelt es sich beim atypischen Vorhofflattern um eine heterogene Gruppe von rechts- und linksatrialen Makro- bzw. Localized-Reentry-Tachykardien, deren kritischer Bestandteil zur Aufrechterhaltung der Tachykardie nicht der cavotrikuspidale Isthmus ist. Atypisches Vorhofflattern tritt gehäuft nach vorangegangener Katheterablation sowie nach herzchirurgischen Eingriffen auf. Die intraprozedurale Erfolgsrate während der Ablation ist hoch, wobei die Rezidivrate von strukturellen Veränderungen der Vorhöfe sowie des zugrundeliegenden Mechanismus abhängig ist. Dieser Artikel bietet einen Überblick über die Mechanismen sowie über Mapping- und Ablationsstrategien der häufigsten Formen von rechts- und linksatrialem atypischem Vorhofflattern. Dieser Beitrag ist Teil der Serie „EP-Basics“ zur gezielten Fortbildung im Bereich *Invasive Elektrophysiologie*. Grundlagen, Klinik und Therapie des atypischen Vorhofflatterns werden mit Fokus auf klinisch relevante Aspekte dargelegt. Vorgehensweise und Befunde der invasiven elektrophysiologischen Diagnostik und die Ablationsbehandlung bilden den Schwerpunkt dieses Artikels.

## Abstract (en)

In contrast to typical atrial flutter, atypical atrial flutter is a heterogeneous group of right and left atrial macro- or localized reentry tachycardias whose critical component for maintaining tachycardia is not the cavotricuspid isthmus. Atypical atrial flutter occurs more frequently after previous catheter ablation and after cardiac surgery. The intraprocedural success rate during ablation is high, although the recurrence rate depends on structural changes in the atria as well as the underlying mechanism. This article provides an overview of the mechanisms as well as mapping and ablation strategies of the most common forms of right and left atrial atypical atrial flutter. This article is part of the “EP Basics” series for targeted continuing education in *invasive electrophysiology*. Basics, clinic and therapy of atypical atrial flutter are presented with focus on clinically relevant aspects. Procedures and findings of invasive electrophysiological diagnostics and ablation treatment are the focus of this article.

Bei atypischem Vorhofflattern handelt es sich um eine Herzrhythmusstörung, die durch eine schnelle, repetitive sowie regelmäßige Depolarisation des atrialen Myokards gekennzeichnet ist und zu atrialen Frequenzen von ca. 300/min (Tachykardiezykluslänge 200 ms) führen kann. In aller Regel kommt es zu einer regelmäßigen Überleitung der Erregung auf die Ventrikel, beispielsweise führen drei atriale Erregungen zu einer ventrikulären Erregung und somit einer 3:1-Überleitung. In seltenen Fällen kann aber auch eine wechselnde Überleitung vorliegen: Die ventrikuläre Frequenz ist dann unregelmäßig, die atriale hingegen regelmäßig [1–3]. Zur Differenzierung zum pseudoregularisierten Vorhofflimmern (beispielsweise bei Patienten unter antiarrhythmischer Medikation) hilft ein Blick auf das Oberflächen-EKG. Hier sollte zur Abgrenzung zum pseudoregularisierten Vorhofflimmern die Morphologie (einheitlich) sowie die Zykluslänge (regelmäßig, meist > 200 ms) der Vorhoferregung berücksichtigt werden (Abb. 1). Jede atriale Makro-Reentry-Tachykardie, an dem der cavotrikuspidale Isthmus mechanistisch nicht beteiligt ist, wird als atypisches Vorhofflattern bezeichnet, sowohl links- als auch rechtsatrial [1–3].

**Figure 1:**
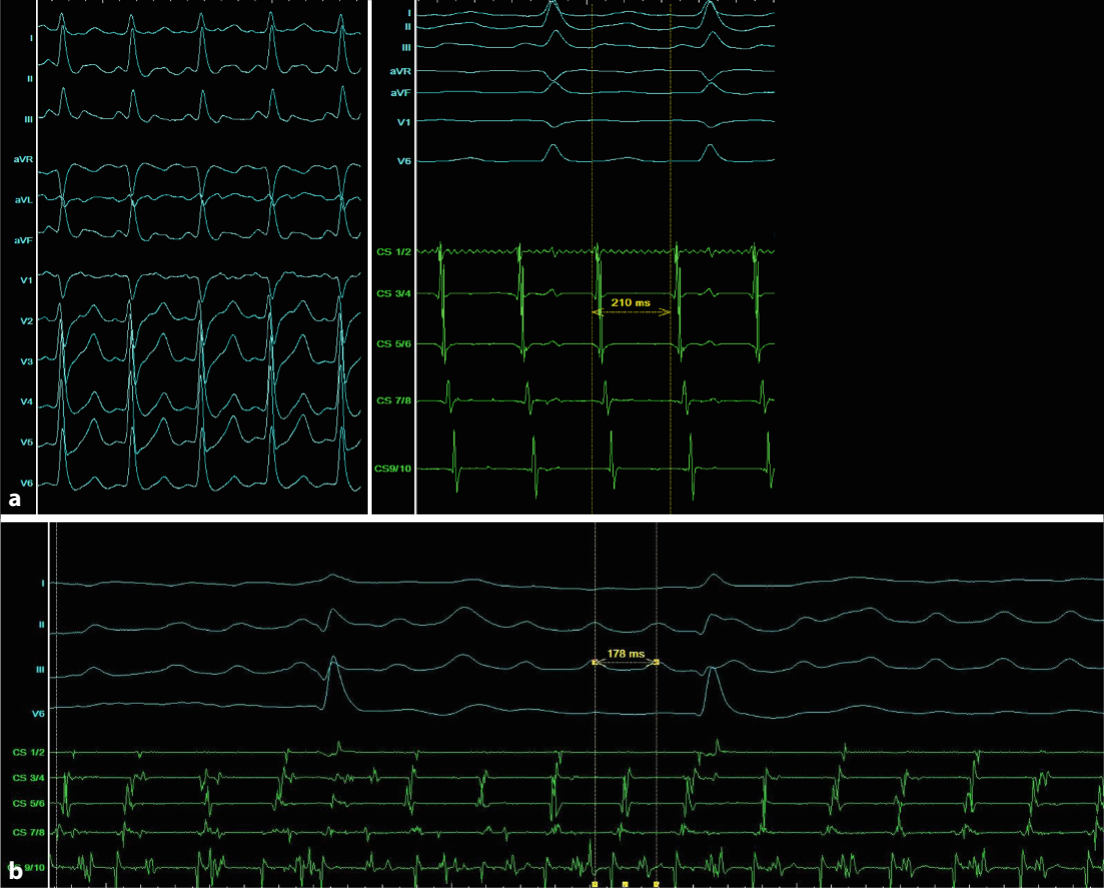

Die Symptomatik der Patienten ist (ähnlich der von Vorhofflimmern) mit Palpitationen, Dyspnoe und Leistungsminderung verbunden. Durch die meist tachykarde Überleitung, die im Vergleich zu Vorhofflimmern schlechter auf eine medikamentöse Frequenzkontrolle anspricht, kommt es bei Patienten mit atypischem Vorhofflattern häufig zu einer deutlichen Symptomatik. Bei atypischem Vorhofflattern besteht, ebenso wie bei Vorhofflimmern, ein erhöhtes Risiko für thrombembolische Komplikationen, so dass in Abhängigkeit der Komorbiditäten (CHADS2VASC2-Score) eine orale Antikoagulation indiziert ist [2]*.*

## Begriffsklärung und Mechanismus

Im Bereich der atrialen Tachykardien gibt es inhaltlich und begrifflich relevante Überschneidungen von (atypischem) Vorhofflattern und fokal-atrialen Tachykardien. Aus Gründen der Didaktik und der Übersichtlichkeit wird hier die verwendete Einteilung benannt. Häufig werden im alltäglichen Sprachgebrauch der Begriff atriale Tachykardie und fokal-atriale Tachykardie synonym verwendet. Dies ist insbesondere im Kontext einer SVT bei strukturell gesunden Vorhöfen der Fall. Im Gegensatz dazu stellt der Begriff der atrialen Tachykardie im Kontext eines strukturell veränderten Vorhofs (vorabladiert, voroperiert oder dilatiert) einen Sammelbegriff von Tachykardien verschiedener Mechanismen dar. Er schließt Reentry-Tachykardien verschiedener Arten mit ein, die z. T. in der Nomenklatur uneinheitlich benannt und gehandhabt werden. Die Einteilung erfolgt hier wir im Folgenden beschrieben.

### Makro-Reentry-Tachykardien

**Figure 2:**
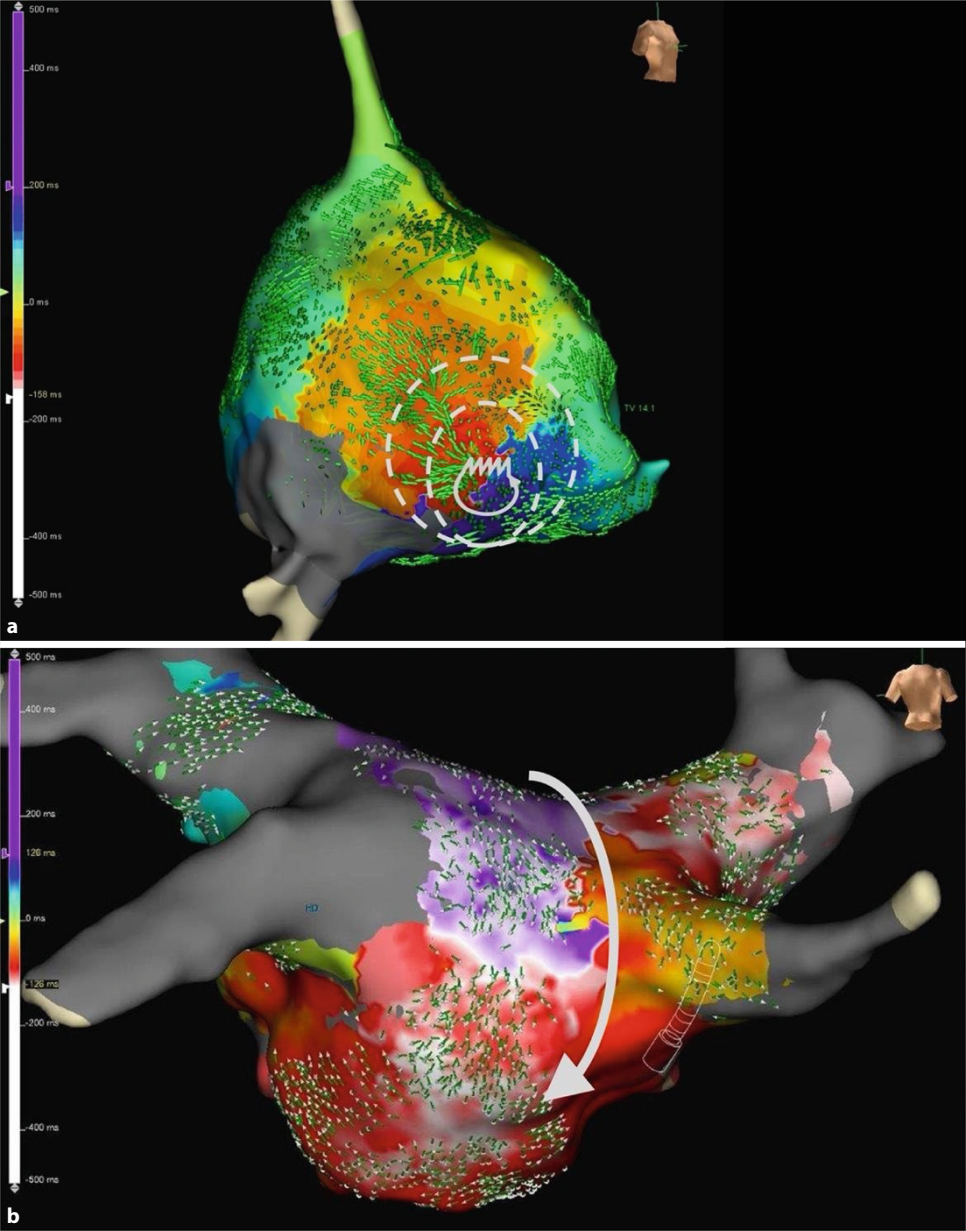

Mechanistisch sind bei Makro-Reentry-Tachykardien (MR-AT) mehrere Segmente des betroffenen Atriums involviert. Das Entrainment ist dann in mindestens 2 Segmenten (beispielsweise dem lateralen und dem anterioren linken Atrium) positiv. Es müssen sich also in zwei unterschiedlichen Segmenten gute Post-pacing-Intervalle zeigen (siehe Abschn. „Entrainment-Mapping“). Im Rahmen des Mappings kann in aller Regel nahezu die gesamte Zykluslänge der Tachykardie entlang der Aktivierungsfront im betroffenen Atrium aufgezeichnet werden ([4]; Abb. 2a).

### Localized-Reentry-Tachykardien

Localized-Reentry-Tachykardien (LR-AT) sind kreisende Erregungen auf einem kleinen Areal (typischerweise von ca. 2 cm^2^), deren Aktivierungsfront sich zentrifugal über das Atrium ausbreitet. Im Rahmen des Entrainments zeigt sich ein positives Post-pacing-Intervall (PPI) lediglich in einem Segment des betroffenen Atriums. Das PPI wird umso kürzer, je näher man dem Ursprung der LR-AT kommt und umso länger je weiter man sich davon entfernt. Hinsichtlich des Mappings können auch hier meist mehr als 75 % der Zykluslänge im betroffenen Atrium abgedeckt werden [5]. Zur genauen Differenzierung bzw. Identifizierung des Ursprungs sind allerdings meist hochauflösende Mappingkatheter notwendig; MR-AT sind (meist) im Aktivierungsmapping einfacher zu identifizieren (Abb. 2b).

### Fokale atriale Tachykardie

Fokale atriale Tachykardien (FAT) sind fokale Erregungen des Vorhofs von einem Punkt (Zellverband) ausgehend. Im Gegensatz zu den vorgenannten Entitäten ist der überwiegende Mechanismus gesteigerte Automatie oder getriggerte Aktivität. Die Erregung des Atriums erfolgt zentrifugal vom Fokus ausgehend. Die gesamte Zykluslänge der Tachykardie ist nicht notwendigerweise im Mapping abbildbar [4].

In dieser Arbeit liegt der Fokus auf den Makro-Reentry-Tachykardien sowie den Localized-Reentry-Tachykardien. Fokale atriale Tachykardien werden in einem gesonderten Kapitel behandelt.

## Ätiologie

Ätiologisch ist atypisches Vorhofflattern eine sehr heterogene Erkrankung und kann durch unterschiedliche atriale Vorerkrankungen begünstigt werden, wohingegen es bei strukturell gesunden Vorhöfen eher eine Rarität darstellt [1]*.* Bei bis zu 15 % der Patienten mit Vorhofflimmern und einer antiarrhythmischen Therapie mit Klasse-Ic- oder Klasse-III-Antiarrhythmika (Propafenon/Flecainid oder Amiodaron) kann es zu einer Regularisierung der atrialen Aktivität kommen, die bis zu einer vollständigen Regularisierung zu atypischem Vorhofflattern reichen kann [1–3].

Das morphologische Korrelat, welches für die Entstehung und Aufrechterhaltung von Reentry-Tachykardien verantwortlich ist wird als *Substrat* zusammengefasst.

Strukturelle Umbauprozesse bedingt durch Myokardfibrose oder iatrogen durch Ablationsläsionen und chirurgische Inzisionen, die zu Vernarbungen führen, dienen zusammen mit anatomischen Strukturen, wie z. B. der Mitralklappen- oder Trikuspidalklappe, als *Substrat* für Reentry-Tachykardien.

Reentry-Tachykardien entstehen dabei oft in Randbereichen von Narben, die oft sehr heterogene Leitungseigenschaften bezüglich der Leitungsgeschwindigkeiten und Refraktärzeiten aufweisen. Voraussetzungen für das Entstehen einer Reentrytachykardie sind:

1. *Unidirektionaler Leitungsblock:* Dieser kann transient auftreten, z. B. verursacht durch eine früh einfallende Extrasystole oder permanent durch Fibrosierungs- bzw. Vernarbungsprozesse, die die Erregungsausbreitung nur in eine Richtung erlauben.
2. *Zone der langsamen Leitung: *Innerhalb von Fibrosierungs/-Narbenarealen finden sich häufig vitale Myozyten mit veränderten Leitungseigenschaften, die als Zonen der langsamen Leitung (sog. Slow-conduction-Zonen) fungieren.
3. *Vorhandensein einer „erregbaren Lücke“:* damit der Reentry-Kreis entstehen bzw. aufrechterhalten werden kann, muss die kreisende Erregung auf nicht refraktäres Gewebe treffen.

**Figure 3:**
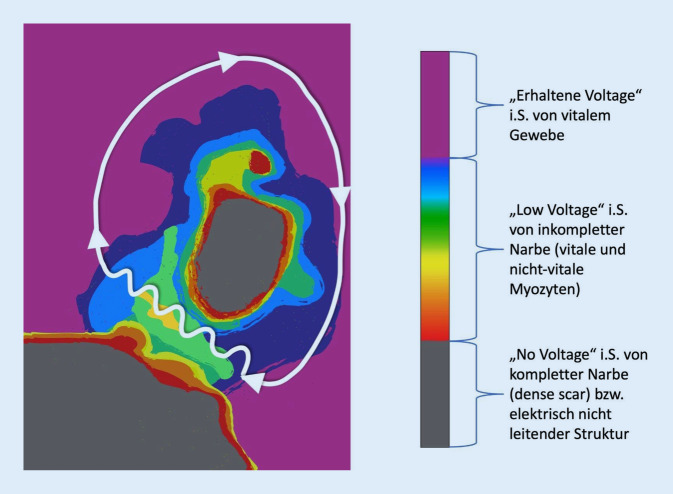

Durch einen unidirektionalen Leitungsblock erregt der elektrische Impuls über eine Zone der langsamen Leitung (z. B.im Randbereich einer Narbe) das gesunde Myokard und trifft im Anschluss auf eine nicht mehr refraktäre Zone der langsamen Leitung, so dass der Reentry-Mechanismus entstehen bzw. aufrechterhalten werden kann (Abb. 3).

Grundsätzlich, wenn auch deutlich seltener, kann auch in *gesundem* Gewebe ein Vorhofflattern auf dem Boden eines Reentry entstehen, eine Slow-conduction-Zone findet man hier nicht, der Reentry kreist dann um ein anatomisches Substrat (meist bei dilatierten Vorhöfen). Im Folgenden sind mögliche Ursachen für das Auftreten von atypischem Vorhofflattern aufgelistet.

### Iatrogenes Substrat nach Ablation oder Inzision

Das zur Entstehung und Aufrechterhaltung der MR-AT notwendige Substrat kann durch vorangegangene Eingriffe (Operation oder Ablation) hervorgerufen werden [1–3]. Häufig kann es nach herzchirurgischen Eingriffen mit Atriotomie zu Vorhofflattern kommen. Etwa nach Verschluss eines atrialen Septumdefekts, einer rechtsatrialen Atriotomie als Zugangsweg für Herzklappenoperationen oder einer MAZE-Operation. Ebenso kann es nach vorangegangenen Ablationen zu atypischem Vorhofflattern kommen.

### Idiopathisches Substrat durch Fibrosierung

Im Rahmen einer ausgedehnten atrialen Fibrosierung, entweder idiopathisch oder nach beispielsweise mediastinaler oder thorakaler Bestrahlung, kann es zu nativem atypischem Vorhofflattern kommen. In einem bipolaren Voltage-Mapping zeigen diese Patienten häufig ausgedehnte Areale mit sog. No- oder Low-Voltage.

### Funktionell

Einige potenziell reversible Faktoren, die zu Vorhofflimmern führen können, führen ebenfalls (wenn auch deutliche seltener) zu Vorhofflattern. Diese sind beispielsweise eine Hyperthyreose, Intoxikationen, Perikarditis, Lungenembolien etc. Häufig zeigen sich die Vorhöfe ohne Low- oder No-Voltage-Areale, so dass nach Beseitigung des Triggers sowie einer Rhythmisierung mittels Kardioversion eine gute Prognose bzgl. der Freiheit von atypischem Vorhofflattern besteht [1–3, 6].

## Aktivierungs- und Entrainmentmapping

### Entrainmentmapping

Das Entrainmentmapping ermöglicht die Diagnose und Charakterisierung von Reentry-Tachykardien. Während das Aktivierungsmapping den zeitlichen Erregungsablauf einer Reentry-Tachykardie darstellt, wird mit dem Entrainmentmapping überprüft, ob sich der Ablationskatheter bzw. diagnostische Elektrodenkatheter innerhalb des Areals der kreisenden Erregung befinden.

Durch die Stimulation mit einer Zykluslänge von 20–30 ms unterhalb der Tachykardiezykluslänge (TCL) wird die Reentry-Tachykardie kurzfristig akzeleriert. Dabei ist sicherzustellen, dass die Reentry-Tachykardie auch tatsächlich auf die Stimulationszykluslänge beschleunigt („gecaptured“) wird (durch Messung der Vorhofzykluslänge beispielsweise im CS-Katheter).

**Figure 4:**
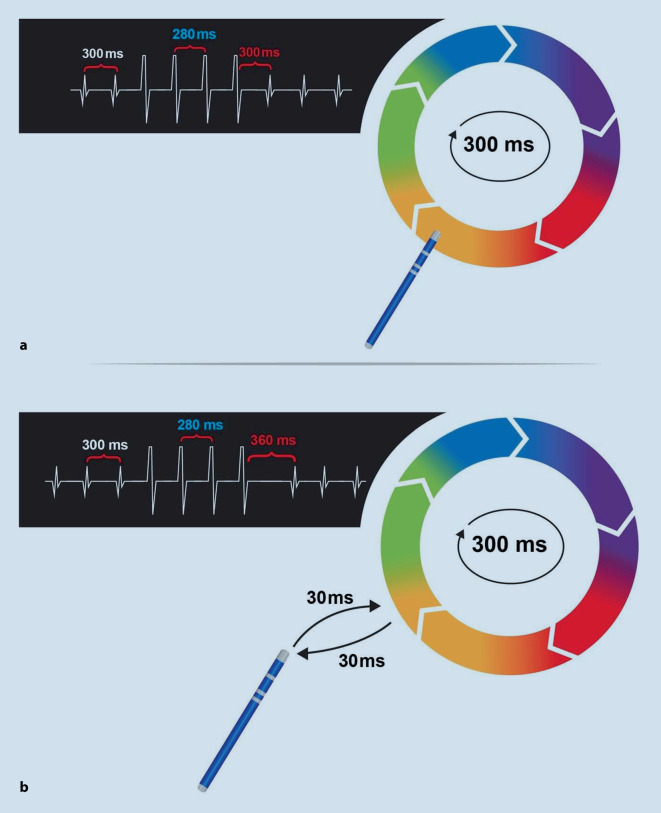

Ob sich der Katheter innerhalb des Reentry-Kreises befindet, lässt sich über das sog. Post-pacing-Intervall (PPI) oder Post-Stimulations-Intervall bestimmen. Als PPI wird das Intervall zwischen der letzten stimulierten Erregung und der ersten nichtstimulierten Aktivierung bezeichnet. Ist dieses Intervall nahezu identisch (< 30 ms über TCL) mit der spontanen Zykluslänge der Tachykardie, so befindet sich der Katheter innerhalb des Reentry. Lange PPI (> 50 ms über TCL) sprechen für eine Katheterlage außerhalb des Reentry-Kreises (Abb. 4).

Die Interpretation des Entrainments kann durch Akzeleration oder Terminierung der Tachykardie erschwert werden. Des Weiteren kann es zu einer Änderung des Tachykardiemechanismus oder zu einer Degeneration in Vorhofflimmern kommen. Es ist daher sinnvoll, nur ca. 20–30 ms unterhalb der Zykluslänge der initialen Tachykardie zu stimulieren bzw. zu entrainen. Als weitere Schwierigkeit tritt manchmal ein fehlendes Capture auf, die Tachykardie lässt sich also nicht auf die stimulierte Zykluslänge akzelerieren. Es sollte deshalb immer von verschiedenen Stimulationsorten „entrained“ werden, dabei ist zu beachten, dass bei Vorhöfen mit starker Fibrosierung und ausgedehnten Low-Voltage-Arealen das Entrainment bzw. das Capturen erschwert sein kann.

### Aktivierungsmapping

Die seit Ende der 1990er Jahre verfügbaren 3‑D-Mappingsysteme sind in der Lage, elektrophysiologische Daten in eine virtuelle dreidimensionale Geometrie der Herzhöhle zu integrieren. Durch Abtasten der Vorhöfe mit einem Ablationskatheter oder mit multipolaren Mappingkathetern wird eine dreidimensionale Geometrie erzeugt und zeitgleich die registrierten Elektrogramme in das 3‑D-Mappingsystem integriert.

Mit dem Aktivierungsmapping lässt sich der zeitliche Ablauf einer Reentry-Tachykardie farbcodiert in einem 3‑D-Mappingsystem darstellen. Für die Erstellung eines Aktivierungsmaps ist es nötig, eine zeitliche Referenz zu definieren, da in einem Makro-Reentry keine *frühen* oder *späten* Signale existieren. Dazu werden in der Regel die Signale im CS-Katheter als zeitliche Referenz definiert. Bezogen auf diese zeitliche Referenz wird ein sog. „Window of interest“ (WOI) definiert, welches die Signale, die früher bzw. später im zeitlichen Bezug zum Referenzsignal auftreten, farbcodiert im 3‑D-Mappingsystem darstellen. Die Länge des WOI sollte in der Regel 90 % der Tachykardiezykluslänge entsprechen und wird begrenzt durch 2 Intervalle: ein Intervall vor der gesetzten Referenz im CS-Katheter und ein Intervall nach dem Referenzsignal im CS-Katheter. Ist das WOI richtig gewählt, zeigt sich im farbcodierten 3‑D-Map bei Reentry-Tachykardien klassischerweise eine sog. *Early-meets-late*-Zone. Des Weiteren lassen sich im Aktivierungsmap die kritischen Zonen der langsamen Leitung (Isthmus), die für die Aufrechterhaltung der Tachykardie notwendig sind, identifizieren.

Für die Erstellung des Aktivierungsmaps können sowohl der Ablationskatheter als auch neue multipolare Mappingkatheter verwendet werden. Der Vorteil von multipolaren Mappingkathetern liegt, neben der schnelleren Akquirierung von Aktivierungspunkten und der hohen örtlichen Auflösung durch die kurze Distanz zwischen den einzelnen Elektroden, in der hervorragenden Signalqualität. Dadurch können auch niedrigamplitudige, fraktionierte Signale sichtbar gemacht werden, die oft charakteristisch für Zonen der langsamen Erregungsleitung (Slow-conduction Zonen) sind [7].

## Häufigste Formen von atypischem Vorhofflattern

Atypisches Vorhofflattern ist sehr heterogen. Bezüglich der Nomenklatur von atypischem Vorhofflattern wird meist versucht, den Laufweg der Aktivierungsfront zu benennen, beispielsweise *perimitral*; die Aktivierung kreist hier um die Mitralklappe. Diese Nomenklatur ist allerdings nur bei MR-AT möglich. Bei LR-AT wird lediglich die anatomische Lokalisation beschrieben, beispielsweise ein LR im Bereich der anterioren Wand des linken Vorhofs. Durch den Einzug von immer besseren Mappingtools mit hochauflösenden Mappingkathetern und optimierten Mappingalgorithmen können LR-AT, aber auch MR-AT immer besser differenziert werden. So kann hierdurch z. B. nach linksatrialer Substratmodifikation oder chirurgischer MAZE-Prozedur eine exakte Identifizierung des Mechanismus erfolgen.

Ein Beispiel: Bei einem Patienten mit atypischem Vorhofflattern nach PVI zeigt sich das Entrainment am Dach des linken Vorhofs sowie im inferioren LA positiv – man würde a. e. von dachabhängigem Vorhofflattern ausgehen. Es könnte sich allerdings bei Z. n. PVI auch um Vorhofflattern um die Pulmonalvenen mit einer Slow-conduction-Zone im Bereich des rekonnektierten Encirclings (dem Zirkel um die Pulmonalvenen) und einer gleichzeitigen Aktivierungsfront über das Dach des LA handeln. Dies wäre nur durch detaillierte Entrainmentmanöver zu identifizieren. Die Anlage einer Dachlinie würde ggf. zu einer Veränderung der AT bis hin zur Terminierung und Konversion in den SR führen; das zugrunde liegende Substrat würde aber ohne Repulmonalvenenisolation nicht adressiert werden, ggf. wäre sogar die Anlage der Dachlinie hier nicht nötig gewesen. Die Kombination aus Entrainment- und hochauflösendem Aktivierungsmapping kann daher sehr hilfreich sein.

Im Folgenden soll eine Übersicht über die häufigsten rechts- und linksatrialen atypischen Vorhofflatternformen gegeben werden (ohne Anspruch auf Vollständigkeit).

Zur genauen Identifikation des Reentry-Mechanismus ist die Verwendung (ultra)-hochauflösenden Mapping-Katheter in Kombination mit Entrainment-Mapping zu empfehlen

**Figure 5:**
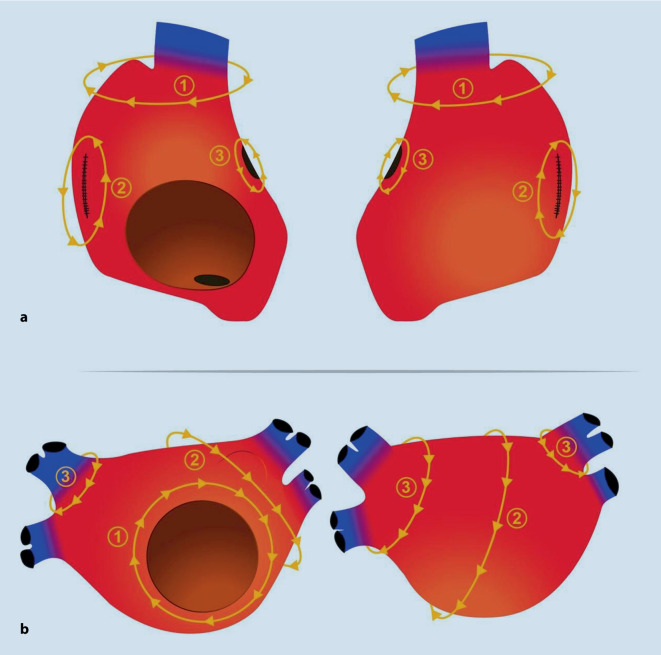

In Abb. 5 ist eine schematische Übersicht der Aktivierungsfronten bzw. des Aktivierungsverlaufs der MR-AT dargestellt. Im Abschnitt „Ablationsstrategie“ soll eine mögliche Ablationslinienführung bzw. Ablationsstrategie aufgezeigt werden.

### Rechtsatriales atypisches Vorhofflattern

#### Upper-Loop-Reentry

##### Mechanismus.

Bei einem Upper-Loop-Reentry zeigt sich eine kreisende Erregung oder Aktivierungsfront um die V. cava superior, zumeist im Uhrzeigersinn. Ein wesentlicher Bestandteil zur Aufrechterhaltung dieses Reentrys ist eine Slow-conduction-Zone im Bereich der Crista terminalis. Der Upper-Loop-Reentry tritt isoliert oder zusammen mit typischem Vorhofflattern auf.

##### CS-Sequenz und Entrainment.

Der Upper-Loop-Reentry zeigt meist eine proximal-distale CS-Sequenz, diese kann allerdings *steiler* sein als bei beispielsweise typischem Vorhofflattern. Ein positives Entrainment findet sich im Bereich der Mündung der SVC sowie im Bereich der superioren Crista terminalis.

##### Ablationsstrategie.

Ablationsziel ist die Schaffung einer linearen Läsion im Bereich des Erregungsdurchtritts in der Crista terminalis. Auf Grund der Nähe zum N. phrenicus ist ein vorheriges Markieren des Verlaufs des Nervs durch „high-output pacing“ dringend zu empfehlen. Bei zu superiorer Ablation muss darauf geachtet werden, den Sinusknoten zu schonen [8].

#### Reentry an der freien Wand des rechten Vorhofs

##### Mechanismus.

Vorhofflattern, welches die freie Wand des rechten Vorhofs einschließt, tritt häufig nach herzchirurgischen Eingriffen auf, bei denen der laterale rechte Vorhof eröffnet wird. Es findet sich jedoch auch bei Patienten ohne vorhergehenden chirurgischen Eingriff. Es zeigt sich eine kreisende Erregung um ein Narbenareal im lateralen rechten Vorhof. Typischerweise findet sich in diesem Bereich eine Linie mit Doppelpotenzialen (als Zeichen eines „local site of block“) und singuläre fraktionierte Areale am unteren bzw. oberen Ende dieses Narbenareals [6].

##### CS-Sequenz und Entrainment.

*Die CS-Sequenz dieses Reentrys verläuft meist von proximal nach distal. *Ein Entrainment von beiden Seiten der Inzision zeigt in der Regel ein Post-pacing-Intervall von < 30 ms. Durch Verlängerung des Narbenareals zur V. cava inferior lässt sich diese Form des Vorhofflatterns mit hoher Erfolgsrate behandeln. Bei Patienten mit typischem Vorhofflattern, die in der Vorgeschichte eine rechtsatriale Atriotomie erhalten haben, ist ein Voltagemap und ggf. Komplettierung der Atriotomienarbe zu empfehlen. Auch hier ist auf Grund der Nähe zum N. phrenicus ein vorheriges Markieren des Nervenverlaufs durch „high-output pacing“ dringend zu empfehlen.

#### Dual-Loop-Reentry

Bei dieser Form handelt es sich um das gleichzeitige Auftreten verschiedener Reentrys. Dies kann eine Kombination aus einem atriotomieabhängigen Vorhofflattern und einem typischen Vorhofflattern sein oder das gleichzeitige Auftreten von einem Upper-Loop-Reentry mit einem narbenassoziiertem Vorhofflattern. Oft führt die Ablation eines Mechanismus zu einer Transition des Vorhofflatterns, die sich oft durch eine Veränderung der Zykluslänge manifestiert und es notwendig macht, den 2. Mechanismus durch weitere Ablationen zu adressieren.

Narbenassoziiertes Vorhofflattern kann auch in anderen Bereichen des rechten Vorhofs als Ausdruck eines *erkrankten* rechten Vorhofs, z. B. bei Erwachsenen mit angeborenem Herzfehler, aber auch nach beispielsweise Kanülierung einer Herz-Lungen-Maschine auftreten. Ablationsstrategie bei diesen Formen des Vorhofflatterns ist es immer, das Narbenareal mit einer weiteren anatomischen Barriere beispielsweise der V. cava inferior/superior durch eine lineare Ablationsstrategie zu verbinden.

### Linksatriales atypisches Vorhofflattern

#### Perimitrales Vorhofflattern

##### Mechanismus.

Bei perimitralem Vorhofflattern zeigt sich eine Aktivierungsfront um die Mitralklappe, entweder im oder gegen den Uhrzeigersinn („clockwise“ oder „counter-clockwise“). Häufig liegt die Slow-conduction-Zone im Bereich der anterioren Wand. Perimitrales Vorhofflattern kann in Kombination mit dachabhängigem Vorhofflattern als Double-Loop-Reentry auftreten.

##### CS-Sequenz und Entrainment.

Die CS-Sequenz kann sich bei korrekter Lage des CS-Katheters proximal-distal (bei einer Clockwise-Aktivierungsfront) oder distal-proximal zeigen (bei Counter-clockwise-Aktivierungsfront). Ein positives Entrainment zeigt sich sowohl im anterioren LA, als auch im lateralen LA.

##### Ablationsstrategie.

Zur Unterbrechung des Reentrys erfolgt meist die Anlage einer Linie vom Mitralanulus zu einer anatomischen Struktur, die nicht leitet, beispielsweise den (isolierten) Pulmonalvenen. Die *Linienführung* erfolgt entweder als anteriore Linie zur LSPV oder RSPV oder als laterale Mitralisthmuslinie zur LIPV. Die Rekonnektionsraten der Mitralisthmuslinie sind auf Grund der anatomischen Dicke des Gewebes mit ca. 70 % im Vergleich zur anterioren Linie mit 40 % deutlich höher. Zusätzlich muss bei ca. 60 % der Patienten über den Koronarsinus eine epikardiale Ablation erfolgen, um die Linie zu blockieren. Die Slow-conduction-Zone liegt meist im Bereich der anterioren Wand, durch die Kombination von Aktivierungsmapping und Voltagemapping kann eine intelligente Linienführung durch No/Low-Voltage-Areale mit Inklusion der Slow-conduction-Zone erfolgen. Im Rahmen der RF-Energieabgabe ist auf Grund der höheren Gewebedicke sowohl im Bereich der anterioren Wand, als auch des Mitralisthmus eine längere Energieabgabe mit mittlerer Power (40–50 W) zu empfehlen. Eine Ethanolinjektion in das Marshall-Venen-Netzwerk zum Erzielen einer Mitralisthmuslinie scheint der alleinigen RF-Ablation der Mitralisthmuslinie überlegen zu sein [9–11].

#### Dachabhängiges Vorhofflattern

##### Mechanismus.

Bei dachabhängigem Vorhofflattern zeigt sich eine Aktivierungsfront, die „über“ das Dach des linken Vorhofs verläuft. Die slow conduction zone liegt häufig im Bereich des postero-superioren linken Vorhofs. Vor allem bei enger anatomischer Beziehung der linken und rechten Pulmonalvenen kann nach PVI mit weit antraler Ablation dachabhängiges Vorhofflattern auftreten.

##### CS-Sequenz und Entrainment.

Die CS-Sequenz kann sich bei korrekter Lage des CS-Katheters (nicht zu weit distal im CS) steil, also mit etwa zeitgleicher Aktivierung der distalen und proximalen CS-Pole zeigen, wenn der CS perpendikulär zur Aktivierungsfront verläuft. Ein positives Entrainment findet sich im Bereich des Dachs des linken Vorhofs sowie im inferioren LA.

##### Ablationsstrategie.

Bei der Ablationsstrategie werden durch die Anlage einer Linie am Dach die Zirkel der linken und rechten Pulmonalvenen verbunden. Dies kann entweder eher anterosuperior oder posterior erfolgen. Bei einer anterosuperioren Linienführung zeigt sich das zu abladierende Gewebe dicker, so dass eine längere Ablationsdauer pro Läsion mit mittelhoher Wattzahl sinnvoll erscheint. Bei posteriorer Linienführung ist das Gewebe dünner, hier darf die anatomische Nähe des Ösophagus und Gefahr einer atrioösophagealen Fistelbildung nicht unterschätzt werden. Eine Ablationsstrategie mit „high power short duration“ (HPSD) kann auf Grund der weniger in die Tiefe reichenden Läsionen hier sinnvoll sein. Häufiger *Rekonnektionsort* der posterioren Dachlinie ist der Übergang zum Ablationszirkel der rechten oberen Pulmonalvene (RSPV).

#### Peri-PV-Vorhofflattern

##### Mechanismus.

Die Aktivierungsfront bei Peripulmonalvenenvorhofflattern läuft um eine oder zwei ipsilaterale Pulmonalvenen. Häufig tritt diese Form von atypischem Vorhofflattern nach inkompletter bzw. rekonnektierter Pulmonalvenenisolation auf, wobei hier die Slow-conduction-Zone häufig im Bereich der undichten Pulmonalvenenzirkel lokalisiert ist. Es besteht eine Überlappung mit dachabhängigem Vorhofflattern.

##### CS-Sequenz und Entrainment.

Die Abgrenzung von dachabhänigem oder peri-PVVorhofflattern kann besonders im Aktivierungsmap schwierig erscheinen, insbesondere, wenn die Bereiche zwischen den ipsilateralen PV nicht sorgfältig gemapped wurden. Es empfiehlt sich daher, grundsätzlich vor Ablation des atypischen Vorhofflatterns rekonnektierte Pulmonalvenen zu reisolieren. Nicht zuletzt deshalb, da isolierte Pulmonalvenen als Konnektionsstelle linksatrialer Linien essenziell sind. Die CS-Sequenz kann je nach betroffenem Pulmonalvenenpaar und Laufrichtung der Aktivierungsfront distal-proximal, proximal-distal oder steil sein.

##### Ablationsstrategie.

Als Ablationsstrategie ist eine zirkumferenzielle Pulmonalvenenisolation zu empfehlen. Es lohnt häufig ein Blick auf Signale *zwischen* den Pulmonalvenen (Carinaregion), da hier Konnektionen (auch epikardial) bestehen können.

#### Septale AT

##### Mechanismus.

Bei septalem atypischem Vorhofflattern stellt die Fossa ovalis die anatomische Begrenzung dar, um die die Aktivierungsfront kreist. Dies kann entweder „clockwise“ oder „counter-clockwise“ erfolgen. Hierbei bilden die rechten Pulmonalvenen sowie der Mitralklappenanulus die funktionellen Barrieren des Reentrys.

##### CS-Sequenz und Entrainment.

Die CS-Sequenz kann bei dieser Vorhofflatterform sowohl distal-proximal, als auch proximal distal sein. Das Entrainment zeigt sich im Bereich des gesamten Septums positiv.

##### Ablationsstrategie.

Häufig terminiert die Tachykardie unter Ablation im Bereich des Septums, bei inkompletter Linie kann diese allerdings relativ einfach reinduziert werden. Es empfiehlt sich daher, eine Ablationslinie zur Konnektion der Fossa ovalis beispielsweise zu den rechten Pulmonalvenen oder dem Mitralanulus. Auf Grund der Dicke des Septums sind längere Ablationszeiten pro Läsion mit mittlerer bis hoher Energie sinnvoll, teilweise ist eine Ablation auch vom rechten Vorhof aus notwendig.

## Ablation von Vorhofflattern

Grundsätzlich ist die Anlage von Ablationslinien, insbesondere im linken Atrium mit hohen Rekonnektionsraten und konsekutiv mit hoher Rezidivrate assoziiert. Des Weiteren sind *undichte* Ablationslinien Nährboden für weitere atriale Arrhythmien. Eine optimale Vorbereitung ist daher unerlässlich für die Behandlung jeglicher Art von atypischem Vorhofflattern. Sinnvoll ist die Verwendung von hochauflösenden Mappingkathetern zur Darstellung der Aktivierungsfront, Slow-conduction-Zonen sowie der Voltage zur Identifikation der besten Ablationsstrategie. Zusätzlich sollten moderne, gekühlte RF-Katheter, wenn möglich mit Anpressdruckmessung und optimierter Spülung, verwendet werden. Zur besseren Stabilisierung sollte die Hinzunahme von (nicht)steuerbaren Schleusen überlegt werden. Nach Anlage von Ablationslinien sollte nach einer adäquaten Wartezeit (in etwa 15–20 min) die bidirektionale Blockierung der Linie mittels „differential pacing“ überprüft werden. Um die Erfolgsrate der Ablation zu erhöhen ist es darüber hinaus sinnvoll, neben einem Blick auf die Voltage (im Hinblick auf die Linienführung), auch auf die (empirische) Wand- bzw. Gewebedicke zu achten. Neuere Ablationsverfahren wie etwa HPSD können hier V. a. in Arealen mit dünnerer Wanddicke (bspw. posteriore Wand des LA) oder kritischen angrenzenden Strukturen (N. phrenicus, Ösophagus) hilfreich sein [10]. Ein weiterer Streitpunkt ist, inwieweit bei MR-AT lediglich die Ablation der Slow-conduction-Zone (soweit identifizierbar) im Gegensatz zur Anlage einer vollständigen Linie genügt. Nach initialer Euphorie eines limitierteren Ablationsansatzes kam es im Verlauf zu mehreren Publikationen, die höhere Rezidivraten im Vergleich zur *vollständigen* Linienanlage gezeigt haben, so dass das limitierte Verfahren der Ablation der Slow-conduction-Zone eine individuelle Entscheidung bleiben sollte [12–14].

## Fazit für die Praxis

- Atypisches Vorhofflattern ist mit einem deutlich erhöhten Risiko für systemische Embolien assoziiert.
- Eine Antikoagulation ist in Abhängigkeit vom CHADS2VAS2-Score empfohlen.
- Atypisches Vorhofflattern ist eine Arrhythmie des strukturell veränderten bzw. voroperierten Vorhofs.
- Grundsätzlich kann atypisches Vorhofflattern auch funktionell oder unter antiarrhythmischer Therapie auftreten.
- Die Radiofrequenzablation ist eine etablierte, effektive und sichere interventionelle Behandlungsmöglichkeit für atypisches Vorhofflattern.
- Die Erfolgsrate der Ablation ist abhängig von der Grunderkrankung bzw. der strukturellen Veränderung der Vorhöfe sowie des zugrundeliegenden Mechanismus.
- Radiofrequenzenergie ist mit gekühlten Kathetern zu bevorzugen, im linken Vorhof ist die Verwendung gespülter Katheter obligatorisch.
- Ziel der Ablation ist die Unterbrechung des Reentrys.
- Makro-Reentry-Tachykardien werden durch Anlage einer Linie zwischen zwei nicht elektrisch leitenden Strukturen behandelt.
- Endpunkt sollte ein bidirektionaler Leitungsblock im Rahmen des „differential pacing“ sein.

## Abkürzungen

FAT: Fokale atriale Tachykardien
HPSD: High power short duration
LR-AT: Localized-Reentry-Tachykardie
MR-AT: Makro-Reentry-Tachykardie
PPI: Post-pacing-Intervall
PVI: Pulmonalvenenisolation
RF: Radiofrequenz
TCL: Tachykardiezykluslänge
WOI: Window of interest
