# Erratum to: Atypical atrial flutter: Diagnostics and therapy

**DOI:** 10.1007/s00399-022-00901-8

**Published:** 2022-10-07

**Authors:** Marc Kottmaier, Felix Bourier, Sonia Busch, Philipp Sommer, Tilman Maurer, Till Althoff, Dong-In Shin, David Duncker, Victoria Johnson, Heidi Estner, Andreas Rillig, Leon Iden, Roland Tilz, Andreas Metzner, K. R. Julian Chun, Daniel Steven, Henning Jansen, Amir Jadidi, Christian Ewertsen, Tilko Reents

**Affiliations:** 1 Abteilung für Elektrophysiologie, Deutsches Herzzentrum München, Technische Universität München, Lazarettstr. 36, 80636 München, Deutschland; 2 Medizinische Klinik, Klinikum Coburg GmbH, Coburg, Deutschland; 3 Klinik für Elektrophysiologie/Rhythmologie, Herz- und Diabeteszentrum NRW, Ruhr-Universität Bochum, Bad Oeynhausen, Deutschland; 4 Klinik für Kardiologie, Asklepios Klinik St. Georg, Hamburg, Deutschland; 5 Med. Klinikum Kardiologie u. Angiologie, Charite – Universitätsmedizin Medizin Berlin, Berlin, Deutschland; 6 Arrhythmia Section, Cardiovascular Institute (ICCV), CLÍNIC – University Hospital Barcelona, Barcelona, Spanien; 7 Klinik für Kardiologie, Herzzentrum Niederrhein, HELIOS Klinikum Krefeld, Krefeld, Deutschland; 8 University Faculty of Health, Center for Clinical Medicine Witten-Herdecke, Wuppertal, Deutschland; 9 Hannover Herzrhythmus Centrum, Klinik für Kardiologie und Angiologie, Medizinische Hochschule Hannover, Hannover, Deutschland; 10 Klinik für Innere Medizin, Universitätsklinikum Gießen, Gießen, Deutschland; 11 Medizinische Klinik und Poliklinik I, LMU Klinikum der Universität München, München, Deutschland; 12 Universitäres Herzzentrum Hamburg, Universitätsklinikum Eppendorf Hamburg, Hamburg, Deutschland; 13 Klinik für Kardiologie, Herz- und Gefäßzentrum Bad Segeberg, Bad Segeberg, Deutschland; 14 Sektion für Elektrophysiologie, Medizinische Klinik II, Universitäres Herzzentrum Lübeck, Universitätsklinikum Schleswig-Holstein (UKSH), Lübeck, Deutschland; 15 Universitäres Herzzentrum Hamburg, Universitätsklinikum Eppendorf, Hamburg, Deutschland; 16 Cardioangiologisches Centrum Bethanien – CCB, Frankfurt, Deutschland; 17 Abteilung für Elektrophysiologie, Herzzentrum der Uniklinik Köln, Köln, Deutschland; 18 Elektrophysiologie Bremen, Bremen, Deutschland; 19 Klinik für Kardiologie und Angiologie, Abteilung für Elektrophysiologie, Herzzentrum Freiburg Bad Krozingen, Bad Krozingen, Deutschland; 20 Klinik für Innere Medizin – Kardiologie und konservative Intensivmedizin, Vivantes Klinikum Am Urban, Berliner-Herzrhythmus-Zentrum, Berlin, Deutschland

**Erratum to:**

**Herzschr Elektrophys 2022**

10.1007/s00399-022-00887-3

Please note that the correct English title should read “Atypical atrial flutter: Diagnostics and therapy”. The original article has been corrected.

